# Global Microarray Analysis of Alkaliphilic Halotolerant Bacterium *Bacillus* sp. N16-5 Salt Stress Adaptation

**DOI:** 10.1371/journal.pone.0128649

**Published:** 2015-06-01

**Authors:** Liang Yin, Yanfen Xue, Yanhe Ma

**Affiliations:** 1 State Key Laboratory of Microbial Resources, Institute of Microbiology, Chinese Academy of Sciences, Beijing, China; 2 University of Chinese Academy of Sciences, Beijing, China; University Paris South, FRANCE

## Abstract

The alkaliphilic halotolerant bacterium *Bacillus* sp. N16-5 is often exposed to salt stress in its natural habitats. In this study, we used one-colour microarrays to investigate adaptive responses of *Bacillus* sp. N16-5 transcriptome to long-term growth at different salinity levels (0%, 2%, 8%, and 15% NaCl) and to a sudden salt increase from 0% to 8% NaCl. The common strategies used by bacteria to survive and grow at high salt conditions, such as K^+^ uptake, Na^+^ efflux, and the accumulation of organic compatible solutes (glycine betaine and ectoine), were observed in *Bacillus* sp. N16-5. The genes of SigB regulon involved in general stress responses and chaperone-encoding genes were also induced by high salt concentration. Moreover, the genes regulating swarming ability and the composition of the cytoplasmic membrane and cell wall were also differentially expressed. The genes involved in iron uptake were down-regulated, whereas the iron homeostasis regulator Fur was up-regulated, suggesting that Fur may play a role in the salt adaption of *Bacillus* sp. N16-5. In summary, we present a comprehensive gene expression profiling of alkaliphilic *Bacillus* sp. N16-5 cells exposed to high salt stress, which would help elucidate the mechanisms underlying alkaliphilic *Bacillus* spp. survival in and adaptation to salt stress.

## Introduction

In their natural habitats, bacteria are often confronted with physicochemical changes in the environment, including osmolarity, pH, temperature, and oxygen concentration [1]; therefore, the ability to adapt to changing and often harsh environments is critical for bacterial survival. The tolerance to salinity and osmotic stress has been studied in a number of bacterial species such as *Escherichia coli* and *Bacillus subtilis* [2,3]. The common strategy used by bacteria to adapt to high salt concentrations is based on the biosynthesis and/or accumulation of organic compatible solutes that do not interfere greatly with the activity of normal enzymes and function as osmoprotectants against high salinity [4,5]. Organic compatible solutes used by various microorganisms include, among others, glycine betaine, proline, trehalose, and ectoine [6–8]. In addition, K^+^ uptake and Na^+^ efflux are also among common mechanisms activated upon exposure to salt stress, resulting in high levels of intracellular K^+^ ions [9,10].

Salt stress also exerts pleiotropic effects on microbial physiology, including changes in membrane composition [11,12], cell wall properties [13], exopolysaccharide structural content [14], cell swarming [15,16], and iron homeostasis [17–19].

Most studies on bacterial adaptation to salt stress have been conducted at neutral or near neutral pH [10,20–22]. In the genus *Bacillus*, salt adaptation of neutral species such as *B*. *subtilis* [10] and *B*. *licheniformis* [5] and alkaliphilic *Bacillus* species such as *B*. *halodurans* [23] and *B*. *pseudofirmus* [24,25] has been studied. A general stress response of *B*. *subtilis* [10] and *B*. *licheniformis* [5] includes osmotically up-regulated genes functionally associated with the synthesis and import of osmostress-relieving compounds (compatible solutes) and the SigB-controlled general stress response. One of the earliest responses of *B*. *subtilis* cell population to different stressful conditions is the immediate induction of a large number of general stress proteins encoded by the sigma B-dependent general stress regulon [26,27]. It has been demonstrated that in *B*. *subtilis*, 37 genes of the SigB regulon are involved in salt adaption because their disruption produces a salt-sensitive phenotype [28]. The SigW and SigM regulons are also induced by salt stress. Similar to that observed with the SigB regulon, disruption of the alternative transcription factor SigM abolishes halotolerance, which suggested that SigM is essential for bacterial survival and growth at high salinity. But this salt sensitiveness might be an indirect phenotype related to the severe cell wall defects exhibited by *sigM* mutants [29,30].

In alkaliphilic *Bacillus* spp., the Na^+^ cycle is critically important for maintaining pH homeostasis. Alkaliphilic *Bacillus* spp. studied to date generally require certain levels of intracellular Na^+^, although the concentration range is species-specific [31]. When Na^+^ concentration is at stressful levels, alkaliphilic *Bacillus* spp. need Na^+^ efflux to keep the intracellular Na^+^ concentration below toxic levels as well as to maintain pH homeostasis. In addition, alkaliphiles face energy problems such as an inverted pH gradient and thus a suboptimal proton motive force [32]. Although certain bioenergetic and structural adaptations to maintain pH homeostasis and intracellular osmotic pressure have been described in *B*. *halodurans* C125 and *B*. *pseudofirmus* OF4, not much is known about the genetic background of these processes [23–25]. Ecophysiological experiments followed by transcriptome analyses should provide additional insights into the molecular mechanisms underlying adaptation of alkaliphilic *Bacillus* species to extreme halo-alkaline conditions [33]. The alkaliphilic *Bacillus* sp. N16-5 is a halotolerant strain isolated from the sediment of Wudunur Soda Lake in Inner Mongolia, China. This strain exhibits an excellent ability to grow over a wide range of pH (8.5–11.5) and NaCl concentrations (0–15%) [34]. In this study, we conducted global transcriptional analysis to investigate the genetic mechanisms underlying the adaptive reactions of alkaliphilic *Bacillus* sp. N16-5 to prolonged growth at different salinities (0%, 2%, 8%, and 15% NaCl) and its responses to a sudden salinity increase from 0% to 8% NaCl.

## Materials and Methods

### Bacterial strain and media

*Bacillus* sp. N16-5 (CGMCC No. 0369) was isolated from the sediment of the Wudunur Soda Lake in Inner Mongolia, China. It was grown aerobically at 37°C and 220 rpm in modified alkaline Horikoshi-II medium containing (g/L): peptone, 5; glucose, 5; K_2_HPO_4_·3H_2_O, 1; Mg_2_SO_4_·7H_2_O, 0.2; yeast extract, 0.1; tricine, 8.96; CAPS, 11.07; CHES, 10.36; and various amounts of NaCl (0, 20, 80 or 150). Medium pH was adjusted to 9.4, using 5 M KOH after sterilization. SA5 medium [35] was used for protoplast regeneration of *Bacillus sp*. N16-5 and neutral complex medium (NCM) [36] was used in deletion mutant construction.

For salt adaption experiments, bacteria were grown overnight in Horikoshi-II medium with different salinity (0%, 2%, 8%, or 15% NaCl). Then the overnight cultures were inoculated respectively to the corresponding fresh Horikoshi-II medium with different salinity (0%, 2%, 8%, or 15% NaCl) at the ratio of 1:100. Samples were collected at early exponential phase (OD_600_ = 0.3).

For salt shock experiments, bacteria were grown in Horikoshi-II medium with 0% NaCl until OD_600_ reached 0.3. Then, 32% (w/v) NaCl stock solution in Horikoshi-II medium was added up to the final concentration of 8% (w/v) NaCl, and samples were collected at 10, 30, 60, and 120 min after salt addition; the sample collected before salt shock (0 min) was used as a control. Cells were immediately precipitated by centrifugation (12,000 × *g*, 1 min, 4°C) and frozen in liquid nitrogen until RNA extraction. The experiments were performed in triplicate.

### RNA isolation and microarray analysis

Frozen cell pellets were grinded in liquid nitrogen to prevent RNA degradation, and total RNA was extracted from homogenized cells using TRIzol reagent (Invitrogen, Carlsbad, CA, USA) according to the manufacturer’s instructions.

Custom-made microarray (8 × 15 K; Agilent Technologies, Santa Clara, CA, USA) consisted of 4,210 60-mer oligonucleotides representing 4,210 ORFs of *Bacillus* sp. N16-5 [37]. Total RNA was checked for the integrity (RIN number) using an Agilent Bioanalyzer 2100 (Agilent), and qualified total RNA was further purified using the RNeasy micro kit (QIAGEN, GmBH, Germany) and RNase-Free DNase Set (QIAGEN). The One-Color Low Input Quick Amp Labeling Kit (Agilent) was used to amplify and label total RNA according to the manufacturer’s instructions, and the labelled cRNA was purified using the RNeasy mini kit. Each microarray was hybridized with 1.65 μg Cy3-labeled cRNA using the Gene Expression Hybridization Kit (Agilent) in a hybridization oven (Agilent), according to the manufacturer’s instructions. After 17-h hybridization, slides were washed in staining dishes (Thermo Shandon, Waltham, MA, USA) with Gene Expression Wash Buffer (Agilent), and scanned using an Agilent Microarray Scanner (G2565CA) at default settings: dye channel, green; scan resolution, 5 μm; PTM, 100% and 10% at 16 bit.

### Microarray data collection and analysis

We used the Feature Extraction software 10.7 (Agilent) to extract and analyse spot intensity in the array. Raw data were normalized using Quantile algorithm in the Gene Spring Software 11.0 (Agilent), and subjected to log_2_ transformation. The genes were marked as A (absent), P (present), or M (marginal) according to signal quality. The microarray data was analysed using the SBC Analysis System (SAS) provided by Shanghai Biochip Co., Ltd (http://www.ebioservice.com). The Student’s *t* test was performed to identify significant differences between the treatments. Fold change was calculated based on the difference in gene expression between two treatments: *fold change* = 2^|*mean*1−*mean*2|^. Fold change could be calculated only when in all three replicate experiments the genes were not flagged ‘A’ for at least one treatment. The genes showing at least 2-fold change in expression (*P* < 0.05) were considered as differentially regulated.

### Real-time PCR

Microarray results were verified by real-time PCR. All gene-specific primers (S1 Table) were designed using the Primer Premier 5 software; 16S rDNA was used as an internal control gene. RNA isolated from bacteria grown at different salinities was reverse transcribed using the PrimeScript RT reagent Kit (TaKaRa, Shiga, Japan), and real-time PCR was performed using the SYBR Premix Ex Taq reagent (TaKaRa). Relative gene expression was calculated according to the 2^−ΔΔ*CT*^ method [38].

### Microarray accession number and gene sequences

Microarray data were deposited in the Gene Expression Omnibus database maintained by the National Center for Biotechnology Information (GEO Series accession number GSE64621) (http://www.ncbi.nlm.nih.gov/geo/). Fold changes in gene transcriptional levels of *Bacillus* sp. N16-5 grown at different salinities versus 0% NaCl are shown in S1 Dataset, and expression changes in response to sudden osmotic shock versus 0-min exposure are shown in S2 Dataset. The sequences of studied genes and proteins are shown in S3 Dataset.

### Construction of Δ*fur* strain

The *E*. *coli/B*. *subtilis* shuttle vector pNNB194 containing a temperature-sensitive *B*. *subtilis* origin of replication [39] was used as the backbone for gene deletion. The mutant Δ*fur* strain was constructed by removing the entire coding sequence of the ferric uptake regulator (*fur*) gene without introduction of an antibiotic resistance gene. In the first step, approximately 1,000-bp DNA fragments located directly upstream and downstream of *fur* were generated and fused by PCR using primers P1, P2, P3 and P4 (S2 Table). The fusion DNA fragment was digested with *Bam*HI and *Sal*I and inserted into pNNB194. The resulting plasmid was introduced into the *Bacillus sp*. N16-5 protoplast as described previously [35], and the transformants were selected by growth on SA5 plates containing 0.5 μg/ml erythromycin at the permissive temperature of 34°C. Single transformant clones were then inoculated into NCM medium supplemented with erythromycin and cultured under aerobic conditions at 34°C in tubes. The cultures were then spread on erythromycin-containing NCM plates and grown at 45°C to select for plasmid integration into the chromosome. To promote homologous recombination in deletion-targeted locus and plasmid elimination, the colonies grown on NCM plates were inoculated into Horikoshi-II medium and grown without selective pressure at 37°C. The cultures were then spread on Horikoshi-II plates, and individual colonies were patched to NCM plates with or without erythromycin. Erythromycin-sensitive colonies were screened by PCR using primers P5 and P6 to identify the *fur* deletion mutant, Δ*fur*.

### Determination of cell density

The growth of the wild-type (WT) and Δ*fur* strains was monitored every 3 h by measuring cell density at 600 nm (OD_600_) in 200-μl aliquots using a 96-well plate spectrometer (SpectraMax 190, Molecular Devices, Sunnyvale, CA, USA).

## Results and Discussion

**Fig 1:**
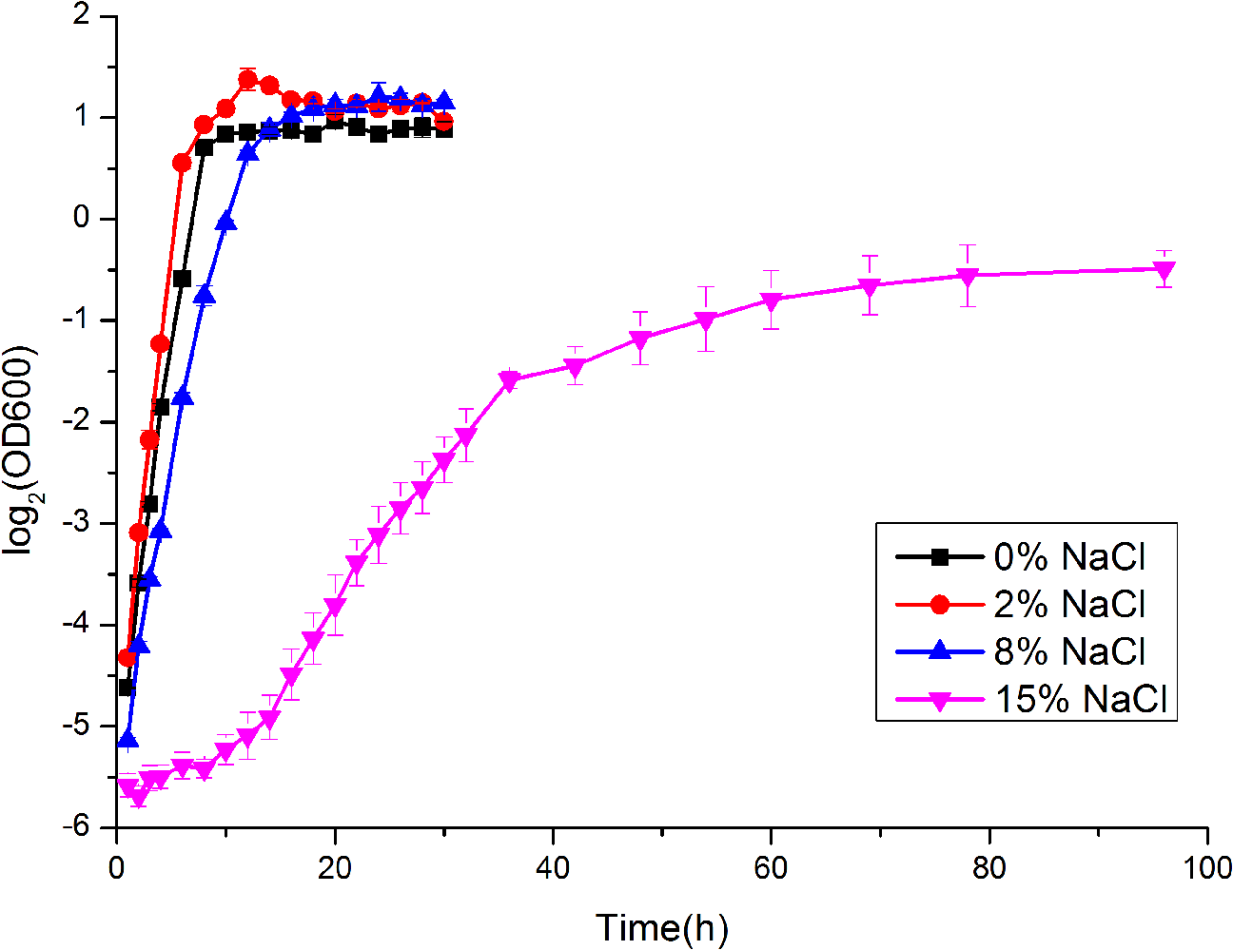
Growth curves for *Bacillus* sp. N16-5 grown in modified alkaline Horikoshi-II medium. *Bacillus* sp. N16-5 cells were cultured in modified alkaline Horikoshi-II medium containing 0%, 2%, 8%, or 15% (w/v) NaCl.

The examination of *Bacillus* sp. N16-5 growth at different salinities showed that the optimal salinity was 2% NaCl (Fig 1). At 15% NaCl, the bacteria demonstrated a long lag phase (about 10 hours). Samples were collected at early exponential phase (OD_600_ = 0.3) for the salt stress test. In salt shock experiments, a sudden increase in salinity resulted in growth arrest for 60 min, after which the growth was resumed (S1 Fig). One-colour microarrays were used to investigate transcriptome profiles of *Bacillus* sp. N16-5 grown at different salinities and under 8% NaCl salt shock. All the replicates showed good repeatability in gene expression signals. The expression data for each gene were compared to that of control (0% NaCl or 0-min exposure), and the detailed results, including fold changes, signal flagging, and statistical significance are shown in S1 and S2 Datasets.

To validate the transcriptome data, eight genes were randomly selected and analysed by real-time quantitative PCR. The analysis indicated that the expression patterns of the eight candidate genes highly correlated with the microarray data (R^2^ = 0.9997/0.9998/0.9996/0.9997/1/1/0.9999) (S2 Fig), demonstrating the reliability of the transcriptome profiling by microarray.

**Fig 2:**
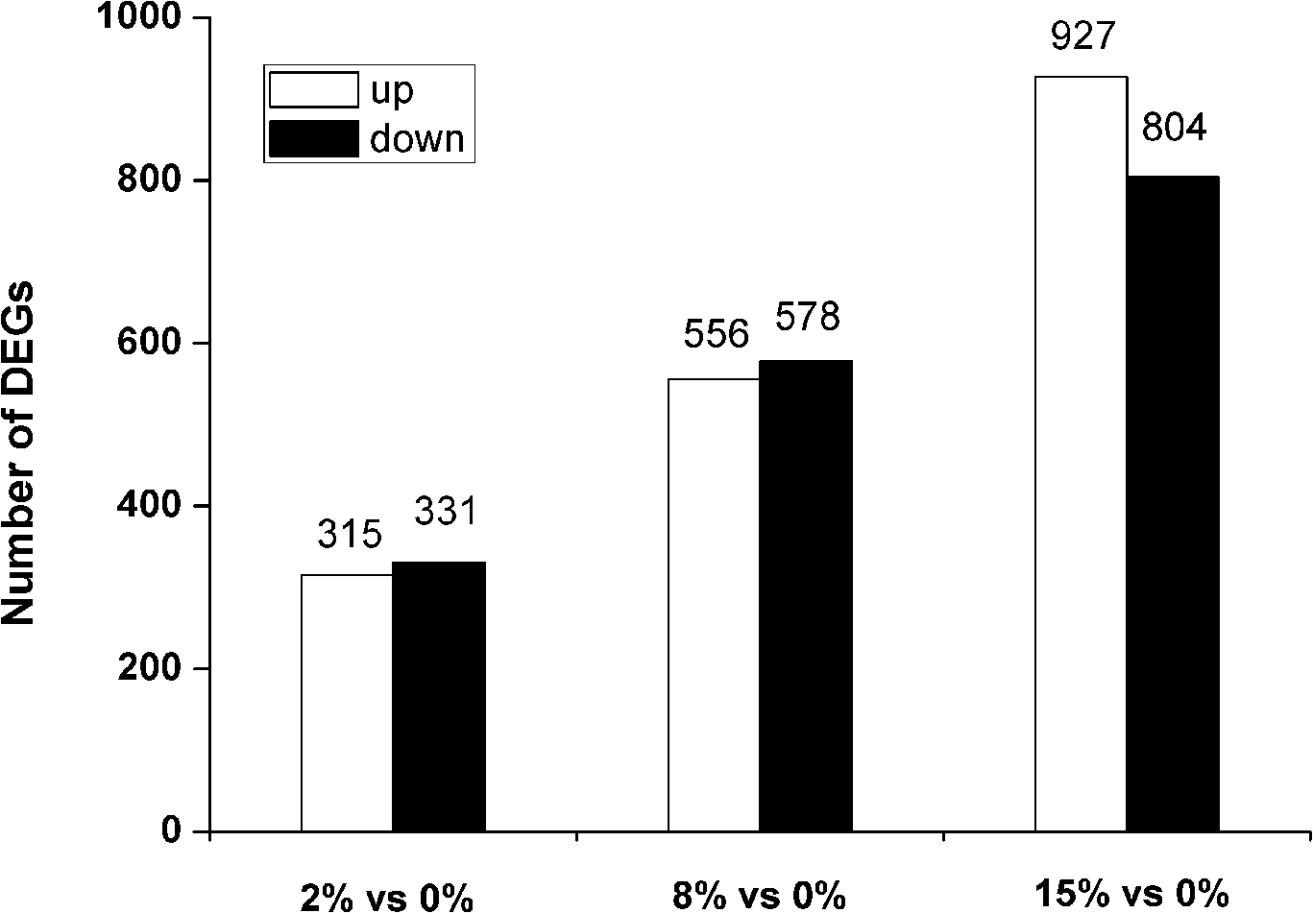
Statistical chart of *Bacillus* sp. N16-5 differentially expressed genes (DEGS) in response to salt stress. Compared to the transcriptional level at 0% NaCl, 315 genes and 331 genes were up-regulated and down-regulated, respectively, at 2% (w/v) NaCl. At 8% (w/v) NaCl, 556 genes and 578 genes were up-regulated and down-regulated, respectively, whereas 927 genes were up-regulated and 824 genes were down-regulated at 15% (w/v) NaCl.

The differential expression of 646, 1134, and 1731 genes was observed in *Bacillus* sp. N16-5 grown at 2%, 8% and 15% NaCl, respectively, compared to control (*P* < 0.05, fold change ≥ 2; Fig 2), indicating that the increase in salinity broadened the spectrum of differentially regulated genes.

### K^+^ uptake and Na^+^ efflux

**Fig 3:**
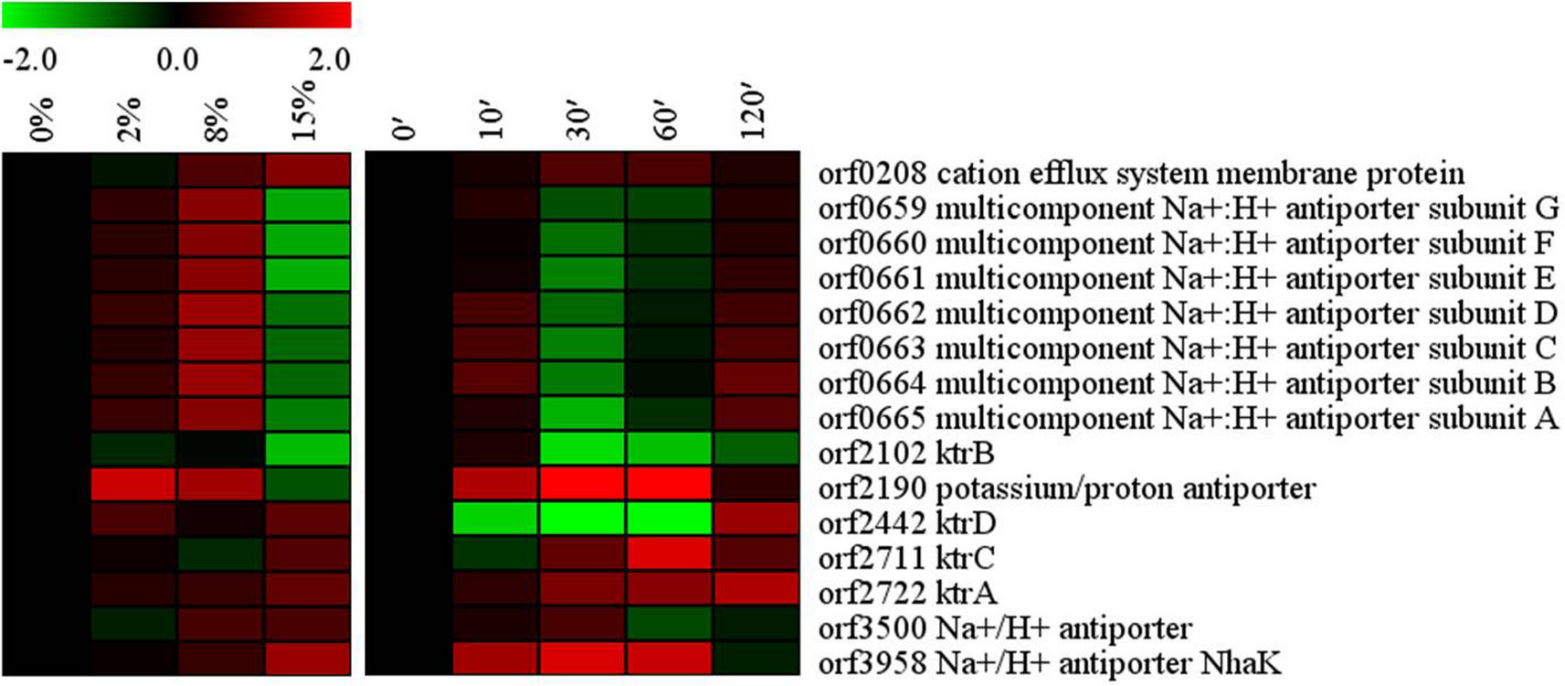
Alterations in K^+^ uptake and Na^+^ efflux related genes. Expression profiles are displayed on the basis of log_2_ ratios.

The transcriptome profiling of *Bacillus* sp. N16-5 growing under high salt conditions identified the genes involved in the maintenance of the intracellular ionic conditions supporting bacterial growth, including the genes encoding K^+^ and Na^+^ transporters (Fig 3). K^+^ uptake transporters (KtrAB and KtrCD) are critical for *B*. *subtilis* against salt stress, but salt stress did not influence the expression of *ktr* [40]. KtrAB (orf2722 and orf2102) and KtrCD (orf2711 and orf2442) are also present in *Bacillus* sp. N16-5. Interestingly, unlike the observation of *B*. *subtilis*, *ktrA* and *ktrB* genes are not in an operon and the expression of *ktrAB* and *ktrCD* were influenced by salt stress in *Bacillus* sp. N16-5. The expression of KtrA increased with the salinity of the medium; and response to salt shock, KtrA showed constant up-regulation during 120 min, KtrC was transcriptionally activated at 30 min. It has been reported that in *B*.*subtills*, the expression of several Na^+^ exporters, including Mrp, NhaK and NhaC, was up-regulated in response to salt stress [10]; but in *Bacillus licheniformis*, the transcription of the genes encoding K^+^ and Na^+^ transporters were not up-regulated in response to salt stress [5]. *Bacillus* sp. N16-5 possesses homologs to some of these transporters (e.g., Mrp and NhaK). The cation efflux system membrane protein (orf0208) and the Na^+^/H^+^ antiporter NhaK (orf3958, 27% amino acid sequence identity to *B*.*subtills* 168 NhaK) in *Bacillus* sp. N16-5 were increasingly expressed with the salinity of the medium, and transcriptionally activated at 30 min and 10 min, respectively, after 8% NaCl salt shock, but at 120 min, the expression returned to the initial levels. These results suggest possible involvement of these transporters in providing *Bacillus* sp. N16-5 halotolerance and salinity stress response. The potassium/proton antiporter (orf2190) and Na^+^/H^+^ antiporter (orf3500), which share 64% and 55% amino acid sequence identity, respectively, with potassium transporter (GI: 763045291) and sodium:proton antiporter (GI: 499511619) from *Bacillus licheniformis*, demonstrated the highest and lowest expression, respectively, at 2% NaCl (the optimal concentration for the growth of *Bacillus* sp. N16-5), suggesting that these transporters may be related to the growth regulation of *Bacillus* sp. N16-5. The Mrp complex formed by the *mrpABCDEFG* operon-coded proteins functions as a Na^+^/H^+^ antiporter playing an important role in *B*. *subtilis* tolerance to salt stress, and the disruption of *mrpABCDEFG* operon confers high Na^+^ sensitivity to *B*. *subtilis* [41]. In *Bacillus* sp. N16-5, the *mrpABCDEFG* operon (orf0659–orf0665) was up-regulated at 2% and 8% NaCl, but down-regulated at 15%. These data may suggest that different Na^+^ transporters play main role at different salinity conditions. Mrp plays main role at 0%-8% NaCl, orf0208 and orf 3958 play main role at 15% NaCl. In salt shock experiments, *mrp* operon showed relatively high transcription at 10 min and 120 min, but low at 30 min and 60 min. Such responses might be related to growth inhibition due to sudden salinity increase. In initial response to salt shock, the bacteria increased Mrp expression, which might be sufficient to cope with high salinity during the lag phase; therefore, further transcription of the operon was suppressed between 30 and 60 min. The up-regulation of Mrp expression at 120 min was consistent with cell growth resumed, when bacteria needed more Mrp molecules for stress response. The results suggest that Mrp may play a role in halotolerance of *Bacillus* sp. N16-5.

### Accumulation of compatible solutes

**Fig 4:**
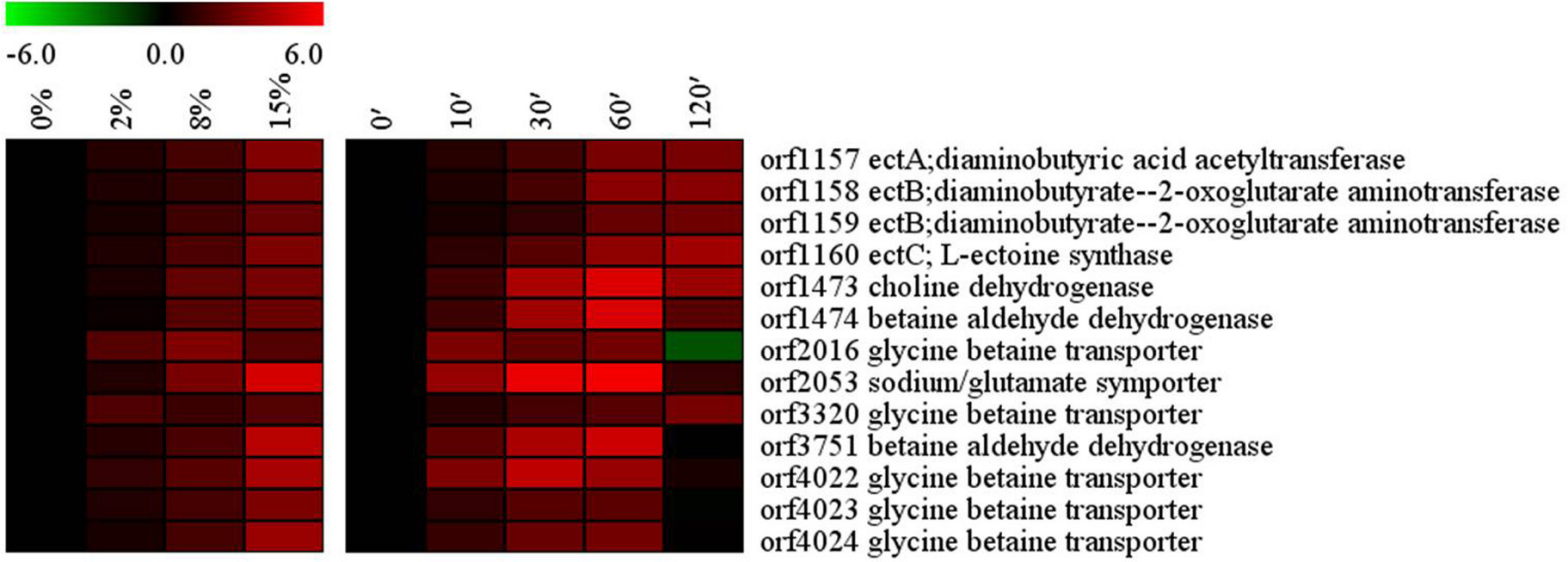
Alterations in compatible solutes related genes. Expression profiles are displayed on the basis of log_2_ ratios.

Microorganisms use organic compatible solutes functioning as osmostress protectants against high salinity. Bacteria can accumulate high levels of compatible solutes either through the uptake from the environment or by the de novo synthesis in high salt conditions [2]. In *Bacillus* sp. N16-5, salt stress induced significant changes in the transcriptional levels of the genes related to the biosynthesis and import of compatible solutes (Fig 4). Under increased salinity, the transcription of the genes encoding glycine betaine transporters OpuD (orf2016 and orf3320), glycine betaine ABC transporter OpuA (orf4022-orf4024), and sodium/glutamate symporter (orf2053) was significantly up-regulated. In *B*.*subtills*, all Opu transporters (OpuA to OpuE) for compatible solute acquisition are up-regulated at the mRNA level at high salinity [10], but the expression of sodium/glutamate symporter GltT was down-regulated at salt stress. It may be because sodium/solute symporters play an important role in Na^+^ re-entry for completion of the Na^+^ cycle in alkaliphilic *Bacillus* species [31]. Similarly, the enzymes choline dehydrogenase (orf1473) and betaine aldehyde dehydrogenase (orf1474 and orf3751), which transform choline to glycine betaine, showed dramatic increase at high salinity. Compatible solute ectione is known as an osmostress protectant widespread among the members of the genus *Bacillus*. Many *Bacillus* possess the ability to synthesize ectoine. The *ectABC* genes encode the diaminobutyric acid acetyltransferase (EctA), the diaminobutyric acid aminotransferase (EctB), and the ectoine synthase (EctC), which constitute the ectoine biosynthetic pathway [42]. In *B*. *pasteurii*, Northern blot analysis demonstrated that the expression of *ectABC* genes is strongly enhanced when the osmolality of the growth medium is raised [43]. The expression of *ectABC* operon (orf1157–orf1160), which encodes EctABC enzymes catalysing the synthesis of ectoine from the precursor l-aspartate-β-semialdehyde, was induced in *Bacillus* sp. N16-5 by high osmolarity. All these genes mentioned above were activated immediately after the shift to high salinity. Among them, glycine betaine transporters (orf2016, orf4022-orf4024) and betaine aldehyde dehydrogenase (orf3751) responded rapidly, showing maximum activation after 30 min and reversing to initial expression level at 120 min, whereas the *ectABC* operon (orf1157–orf1160) showed high transcriptional activity up to 120 min. These data indicate that *Bacillus* sp. N16-5 genes related to compatible solute transport and synthesis show rapid transcriptional activation in response to high salinity. In this haloprotective mechanism, glycine betaine may be the compatible solute important for the immediate reaction to salt stress, whereas ectoine may be involved in long-term halotolerance.

### Cell envelope and cell wall

**Fig 5:**
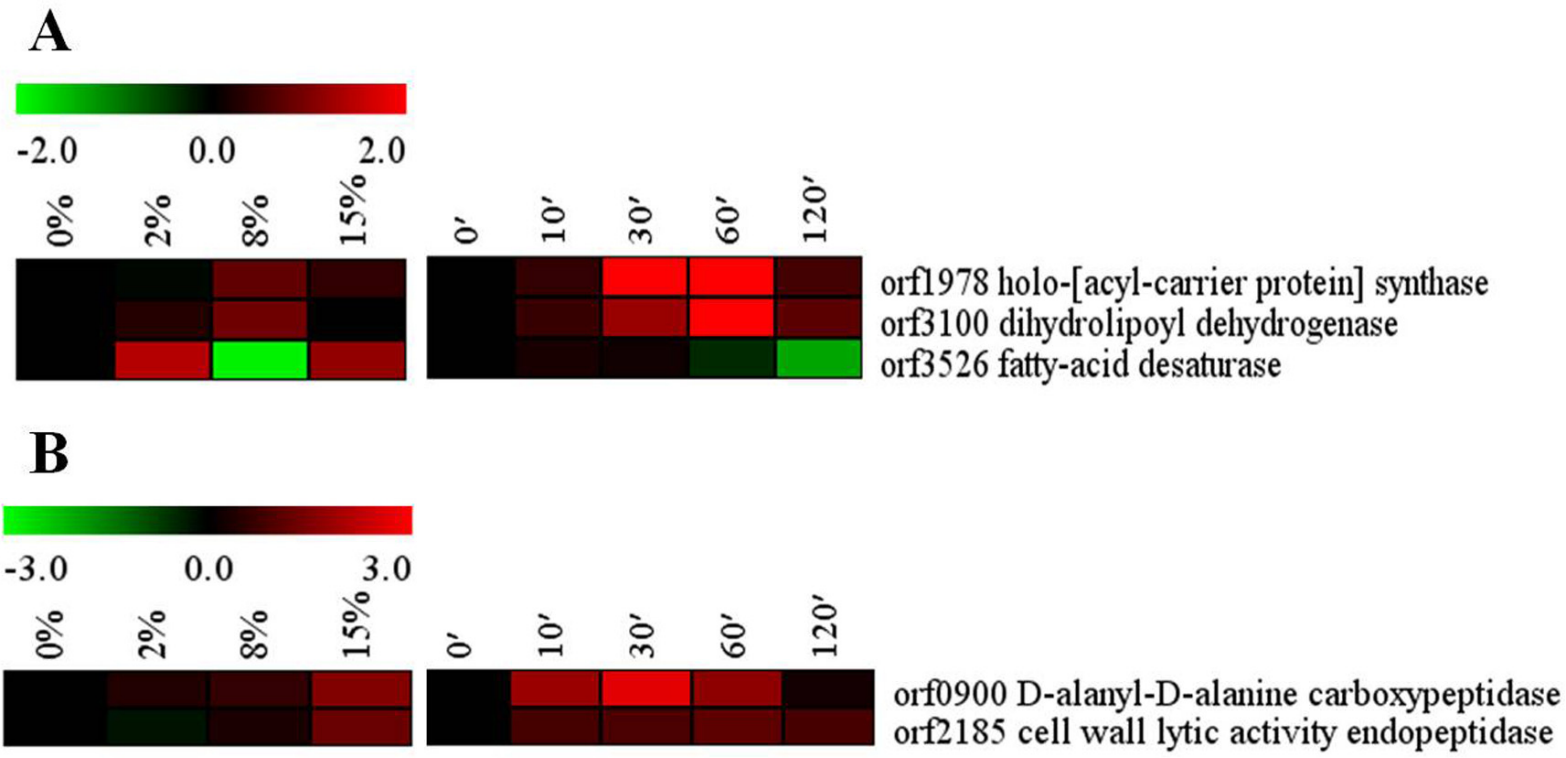
Alterations in cell envelope and cell wall related genes. (A) cell envelope related genes; (B) cell wall related genes. Expression profiles are displayed on the basis of log_2_ ratios.

Most enzymes involved in fatty acid and lipid synthesis were transcriptionally repressed by high salinity (S1 and S2 Datasets). However, the enzymes responsible for the branch-chain fatty acid synthesis, including dihydrolipoyl dehydrogenase (orf3100) and holo-[acyl-carrier protein] synthase (orf1978) were found slightly induced (Fig 5A). Moreover, the expression of the *des* gene (orf3526), which encodes fatty acid desaturase, was also increased. These results indicate that the synthesis of branch-chain and unsaturated fatty acids is increased in *Bacillus* sp. N16-5 exposed to high salinity. The adaptation of *B*. *subtilis* to high salt conditions is commonly accompanied by the changes in the composition of the cell membrane, especially in its fatty acid and lipid components [13,15]. Thus, it has been reported that the increase in the content of cardiolipin, and saturated straight-chain and unsaturated fatty acids may correlate with higher salt resistance of *B*. *subtilis* [12], suggesting that fatty acid metabolism may be involved in *Bacillus* sp. N16-5 response to salt stress. Along with possible rearrangements in the cell membrane, high salinity appeared to affect *Bacillus* sp. N16-5 cell wall (Fig 5B); thus, the genes encoding penicillin-binding protein d-alanyl-d-alanine carboxypeptidase (orf0900) and cell wall lytic activity endopeptidase (orf2185) were up-regulated. In *B*. *subtilis*, penicillin-binding protein Pbp4* (*pbp*E), an endopeptidase that cleaves peptidoglycan cross-links, was induced by high salinity, while *pbpE* disruption led to a salt-sensitive phenotype [10,44]. Thus, the orf0900 and orf2185 genes may play an important role in cell wall rearrangements during salt adaptation of *Bacillus* sp. N16-5.

### Molecular chaperones

**Fig 6:**
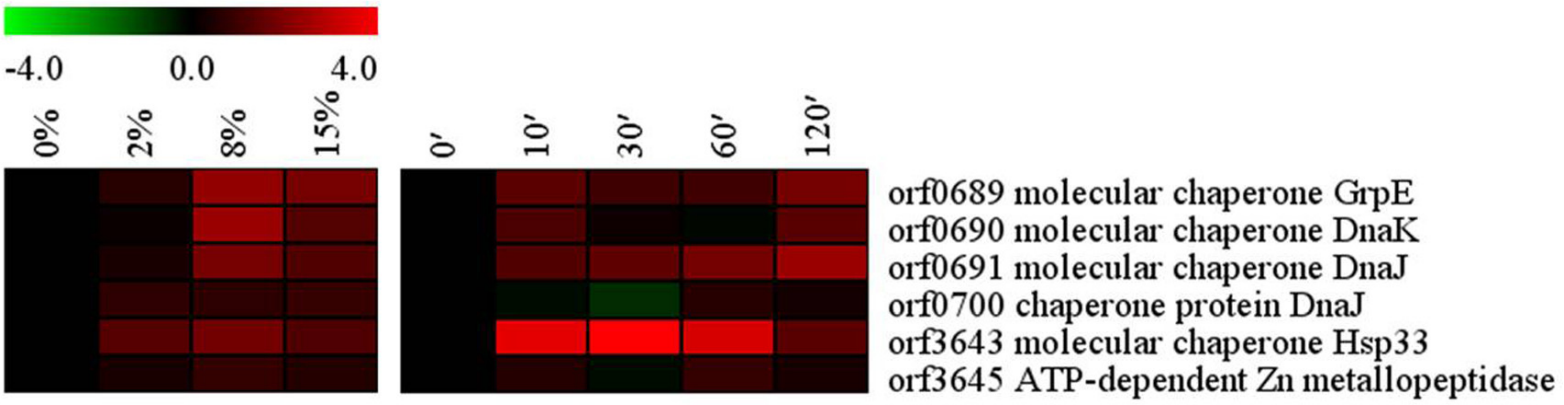
Alterations in molecular chaperones related genes. Expression profiles are displayed on the basis of log_2_ ratios.

Molecular chaperones play an essential role in many cellular processes by stabilizing other proteins’ conformation and refolding of proteins affected by environmental stress, thus acting as stress response factors [45]. In *Bacillus* sp. N16-5, the chaperone-encoding genes *grpE* (orf0689), *dnaK* (orf0690), *dnaJ* (orf0691, orf0700), and *hslO* (orf3643) were up-regulated in high salinity environment (Fig 6). In addition, the ATP-dependent metallopeptidase *ftsH* gene (orf3645) showed slightly increased transcription. The transcription level of molecular chaperon-encoding orf0689–0691 and orf3643 (Hsp33) was dramatically increased 10 min after the salt shock, while orf0700 and orf3645 showed scarce response. Hypersalinity affects protein structure and may induce protein denaturation and misfolding, suggesting that transcriptional activation of molecular chaperones could promote stabilization and refolding of the denatured proteins, while FtsH could degrade the misfolded proteins.

### SigB and SigB regulon

**Fig 7:**
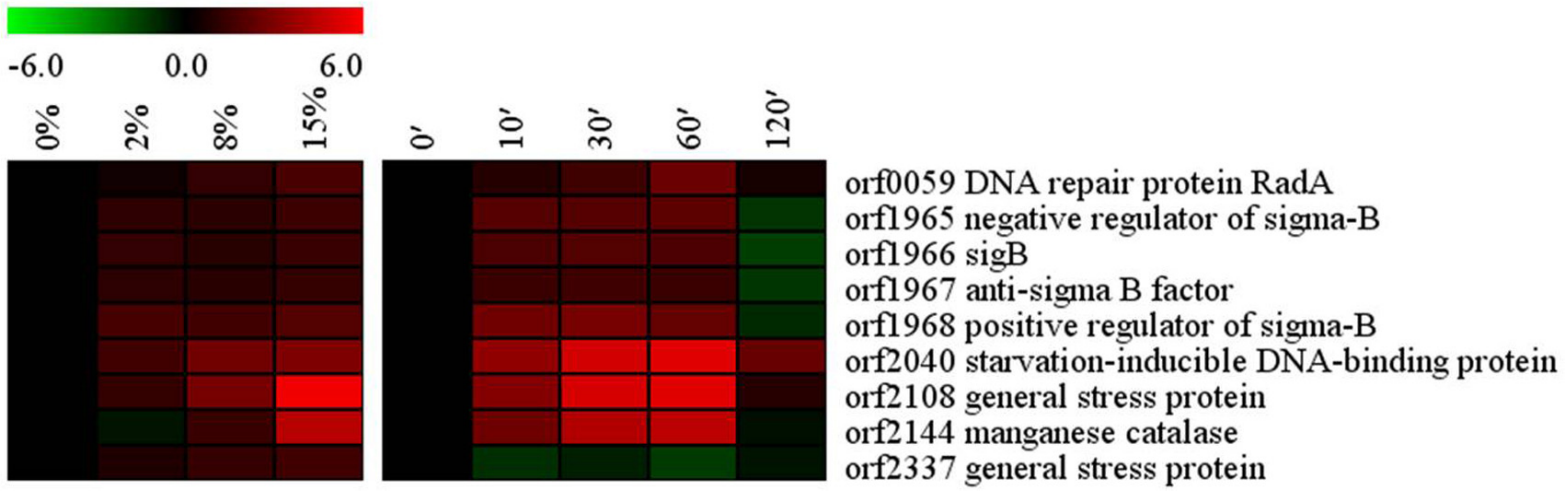
Alterations in SigB and SigB regulon related genes. Expression profiles are displayed on the basis of log_2_ ratios.

The SigB operon, which encodes an alternative sigma factor SigB (orf1966), negative regulator of sigma B (orf1965), anti-sigma B factor (orf1967), and a positive regulator of sigma B (orf1968) was up-regulated when the strain was cultured at 2%, 8% and 15% NaCl (Fig 7), indicating a relationship of SigB to *Bacillus* sp. N16-5 halotolerance. The general stress factor SigB contributes to the ability of microorganisms such as *B*. *subtilis*, *Listeria monocytogenes*, and *Staphylococcus aureus* to survive under environmental and energy stress conditions [46–50]. In *B*. *subtilis*, 37 genes of the SigB regulon have an important role in haloadaption since the disruption of these genes results in a salt-sensitive phenotype [28]. In *Bacillus* sp. N16-5, some homologues of these genes, including general stress protein (orf2108 and orf2337), DNA repair protein RadA (orf0059), starvation-inducible DNA-binding protein (orf2040), and catalase (orf4159 and orf2144) were also up-regulated at high salinity. It has been reported that microorganisms challenged with osmotic shock acquire certain resistance to high temperature and oxidative stress [20]. Our data also indicate that salt stress might induce *Bacillus* sp. N16-5 general stress response system, including DNA base excision repair, mismatch repair, and homologous recombination. The SigB regulon also showed rapid response to hypersalinity shock with most of the genes being induced 10 min after salt addition. The rapid response of global regulatory system could enable *Bacillus* sp. N16-5 to initiate the transcription of the genes involved in providing tolerance to sudden environmental changes.

### Flagellar system

**Fig 8:**
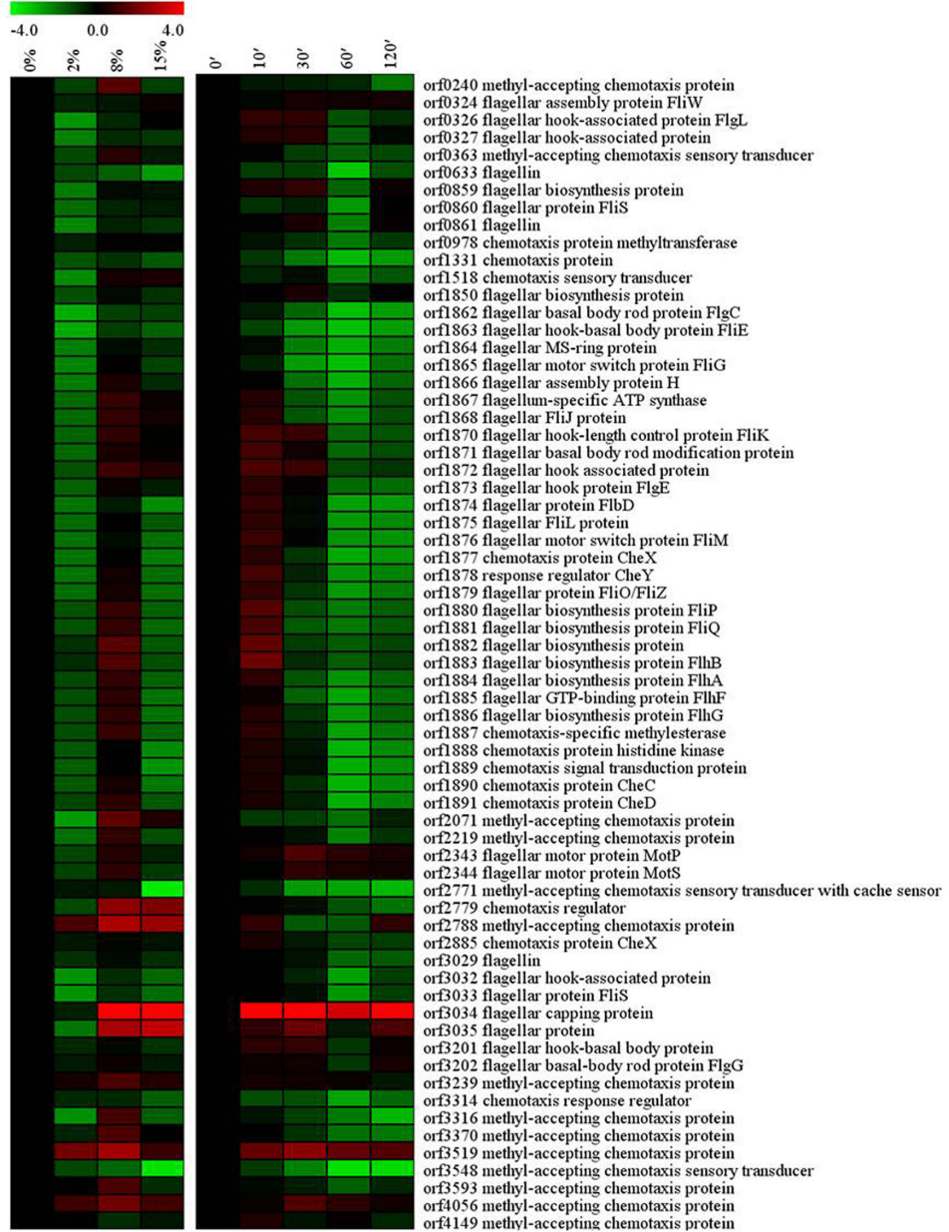
Alterations in flagellar system related genes. Expression profiles are displayed on the basis of log_2_ ratios.

In *B*. *subtilis*, high salinity repressed the genes regulating chemotaxis and motility, which can severely impair cell swarming ability [15]. It has been reported that the down-regulation of flagella-related genes might be necessary for *Shewanella algae* to conserve energy for Na^+^ transport in hypersaline conditions [16]. There are 66 genes possibly involved in chemotaxis and cell motility in *Bacillus* sp. N16-5 (Fig 8). Among these genes, 13 genes were down-regulated, whereas a few genes were up-regulated by high salinity, a few genes were up-regulated at both 8% and 15% NaCl and 19 genes were induced at 8% NaCl, but repressed at 2% and 15% NaCl. This phenomenon may be caused by following several reasons: (1) In alkaliphilic *Bacillus* species, the Na^+^ cycle facilitates Na^+^ re-entry, possibly via ion channels associated with the Na^+^-dependent flagellar motor [31]. Therefore, *Bacillus* sp. N16-5 would down-regulate the expression of flagella-related genes as an adaptation mechanism to reduce Na^+^ re-entry and maintain the intracellular ion homeostasis in high alkaline and salinity conditions. (2) Different flagellin genes are transcribed with different sigma factors. For example, in *Helicobacter pylori*, two flagellin genes, *flaA* and *flaB* are regulated by two different sigma factors (σ^28^ and σ^54^, respectively), suggesting that these genes may be differently expressed depending on environmental conditions [51]. Therefore, different flagellin genes may have different transcription levels at salt stess. (3) Different methyl-accepting chemotaxis proteins (MCPs) respond to different environmental signals, such as Tar (taxis toward aspartate and maltose, away from nickel and cobalt), Tsr (taxis toward serine, away from leucine, indole, and weak acids) in *Escherichia coli* [52]. The up-regulated MCPs may respond to salt stress.

### Iron homeostasis

**Fig 9:**
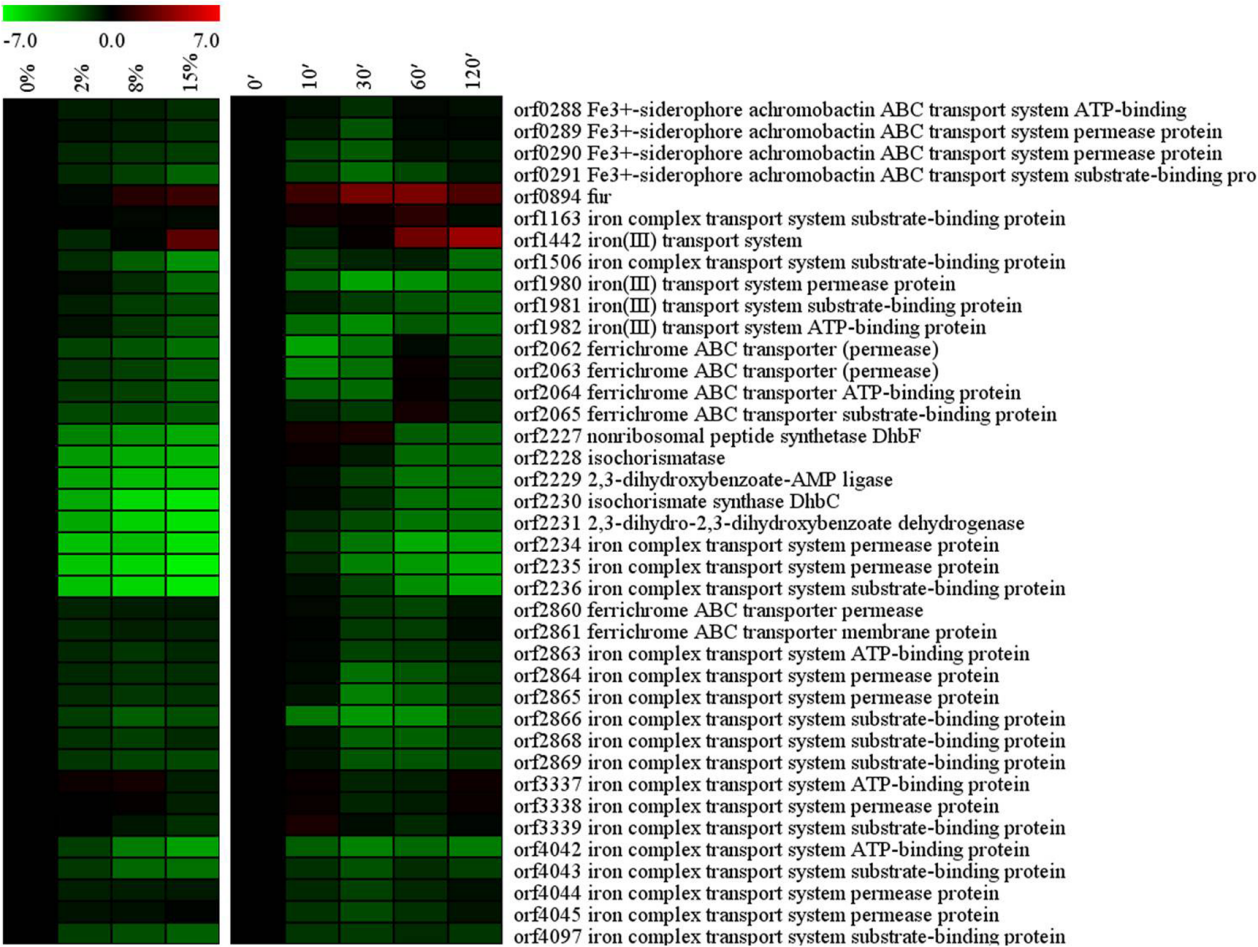
Alterations in iron homeostasis related genes. Expression profiles are displayed on the basis of log_2_ ratios.

**Fig 10:**
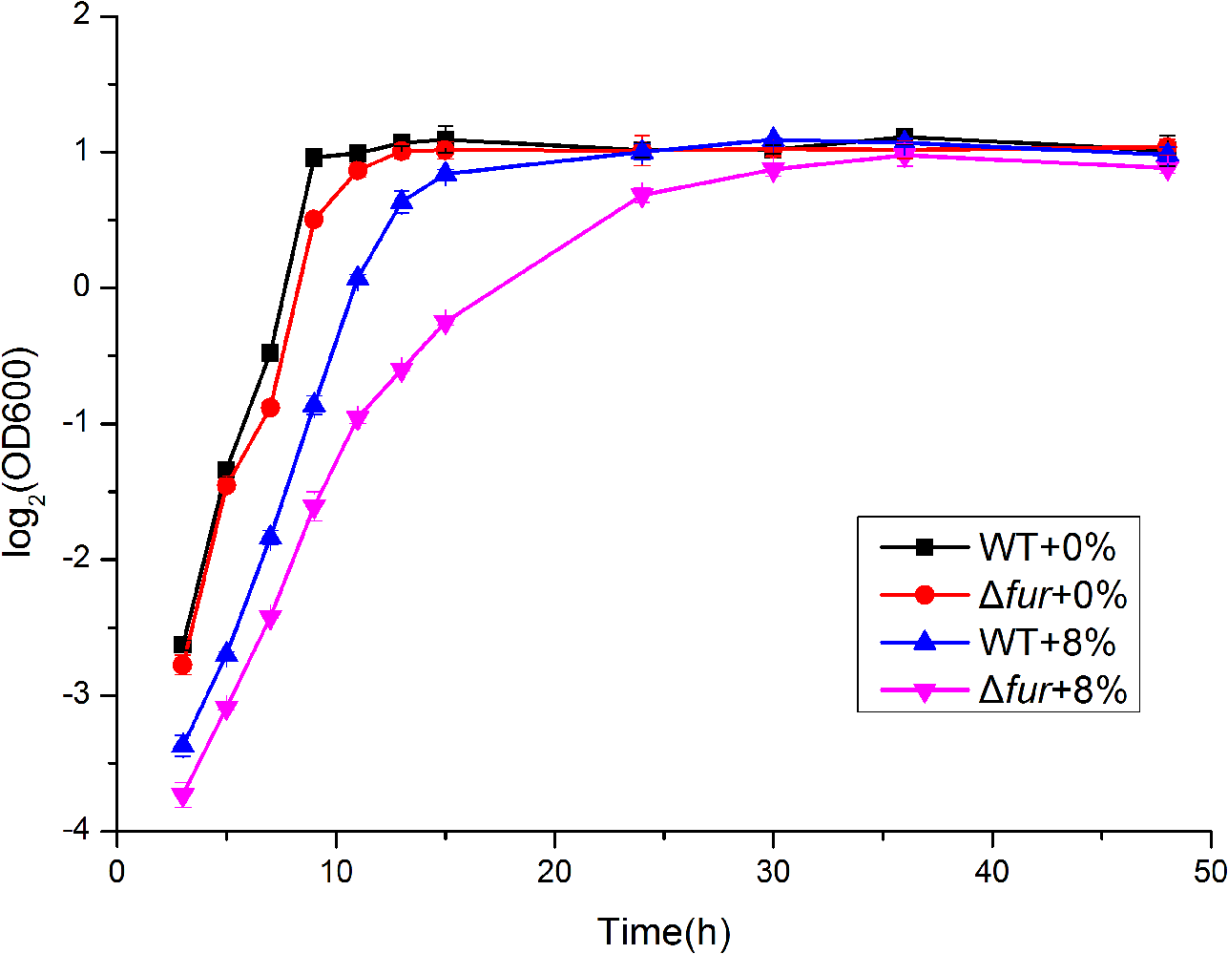
Growth curves of WT and Δ*fur* at different salinities. The WT strain and Δ*fur* strain were cultured in modified alkaline Horikoshi-II medium containing 0% or 8% (w/v) NaCl.

In *B*. *subtilis*, high salinity could induce iron limitation [17]. Consistent with this notion, high salt concentrations affected iron homeostasis in *Bacillus* sp. N16-5 (Fig 9). Ferric uptake regulator (Fur, orf0894) is the key regulator of iron homeostasis controlling the expression of many genes in response to iron availability. Hypersalinity up-regulated *fur* transcription in *Bacillus* sp. N16-5 and significantly down-regulated the iron uptake-related genes negatively controlled by Fur, including the *dhbACEBF* operon (orf2227–orf2231), which encodes the enzymes involved in the synthesis of siderophore, ferrichrome ABC transporter system (orf2062- orf2065) and Fe^3+^-siderophore achromobactin ABC transport system (orf0288- orf0290) and others. These finding suggest that in *Bacillus* sp. N16-5, iron requirement is lower at high salinity. Although our results are different from the findings in *B*. *subtilis* [17], they are consistent with the data on the halophilic bacterium *Chromohalobacter salexigens* [18] and *Helicobacter pylori* [19], where iron requirement is also lower at high salinity. In *Chromohalobacter salexigens*, Fur has been implicatedin the genetic control of the *ect*ABC gene expression in high salt conditions [18], and in *Helicobacter pylori*, Fur isshowed to be essential for the growth in hypersaline conditions [19]. In this study, the functions of the *fur* gene were investigated in the mutant *Bacillus* sp. N16-5 strain Δ*fur* obtained by *fur* knockout through homologous recombination. The growth curves of the WT and mutant Δ*fur* strains were similar at 0% salinity (Fig 10). At 8% salinity, the mutant strain exhibited slower growth, but eventually reached the same cell density at the stationary phase, indicating that the *fur* gene is important but not essential for the growth of *Bacillus* sp. N16-5 at high salinity. The function of Fur in *Bacillus* sp. N16-5 response to salt stress is still unclear. These data suggest that the iron homeostasis regulator Fur may function as a part of the complex circuit that controls the response of *Bacillus* sp. N16-5 to salt stress.

## Conclusions

In this study, we performed comprehensive transcriptomics analysis of the mechanisms underlying the adaptation of alkaliphilic *Bacillus* sp. N16-5 to high salt stress. A broad spectrum of genes involved in ion transport, compatible solute accumulation, cell wall and membrane formation, protein structure stabilization, and general stress response were up-regulated by high salinity, indicating that *Bacillus* sp. N16-5 halotolerance is based on global cooperation of various factors rather than on an individual protein. Most of the detected genes changing in gene expression were also trigged by salt shock, suggesting the role of these genes both in salt stress response and halotolerance, which is a pattern different from that reported for *B*. *subtilis*, where most of the genes that were immediately regulated by salt shock did not show significant differences during continuous salt exposure [15].

In this study, we also provided transcriptional profiling of the dynamic changes induced in *Bacillus* sp. N16-5 by hypersalinity shock. Time-dependent variations in the expression of different genes suggest differential regulation and functional response of the encoded proteins, which may act in concert in providing *Bacillus* sp. N16-5 adaptation to high salt conditions. The specific regulatory mechanisms involving the identified genes require further research.

The presented global transcriptional analysis of alkaliphilic halotolerant *Bacillus* sp. N16-5 can help elucidating the mechanisms underlying microbial survival and adaptation to salt stress.

## Supporting Information

S1 Dataset Results of *Bacillus* sp. N16-5 grown at different salinities microarray data analysis. Different salinities vs 0% NaCl (>2-fold): the fold changes in the transcription levels of genes for the strain grown on specific salinity versus 0% NaCl (Genes that were differently transcribed by at least 2-fold and have *P*-value < 0.05 were considered); different salinity versus 0% NaCl (full): the log_2_ transformed normalization signals and the flags of signal of each biological replicate under each condition, the fold changes in the transcription levels of genes for the strain grown on the specific salinity versus 0% NaCl and the *t*-test results. (XLSX)
(XLSX)

S2 Dataset Results of *Bacillus* sp. N16-5 salt shock microarray data analysis. Different time vs 0 min (>2-fold): the fold changes in the transcription levels of genes for the strain grown at different time after salt shock versus 0 min (Genes that were differently transcribed by at least 2-fold and have *P*-value < 0.05 were considered); different time versus 0 min (full): the log_2_ transformed normalization signals and the flags of signal of each biological replicate under each condition, the fold changes in the transcription levels of genes for the strain grown at different time after salt shock versus 0 min and the *t*-test results. (XLSX)
(XLSX)

S3 Dataset Gene and protein sequences involved in this study. (TXT)
(TXT)

S1 Fig Effect of salt shock on growth of *Bacillus* sp N16-5. Bacteria were grown in Horikoshi-II medium with 0% NaCl until OD600 reached 0.3. Then, 32% (w/v) NaCl stock solution in Horikoshi-II medium was added up to the final concentration of 8% (w/v) NaCl, and samples were collected at 10, 30, 60, and 120 min after salt addition; the sample collected before salt shock (0 min) was used as a control. (TIF)
(TIF)

S2 Fig Validation of the DNA microarray results by RT-PCR. Eight genes were selected at random from differentially expressed genes and their expression levels were assessed by RT-PCR. (A) 2% vs 0%. (B) 8% vs 0%. (C) 15% vs 0%. (D) 10 min vs 0 min.(E) 30 min vs 0 min.(F) 60 min vs 0 min.(G) 120 min vs 0 min. (TIF)
(TIF)

S1 Table The primers used for Quantitative RT-PCR amplifications (DOCX)
(DOCX)

S2 Table The primers used for construction of Δ*fur* strain (DOCX)
(DOCX)
