# Supplementary figures and images for: Global Microarray Analysis of Alkaliphilic Halotolerant Bacterium Bacillus sp. N16-5 Salt Stress Adaptation
Source: PLoS One. 2015 Jun 1;10(6):e0128649. doi: 10.1371/journal.pone.0128649 (PMC4452262; doi:10.1371/journal.pone.0128649)

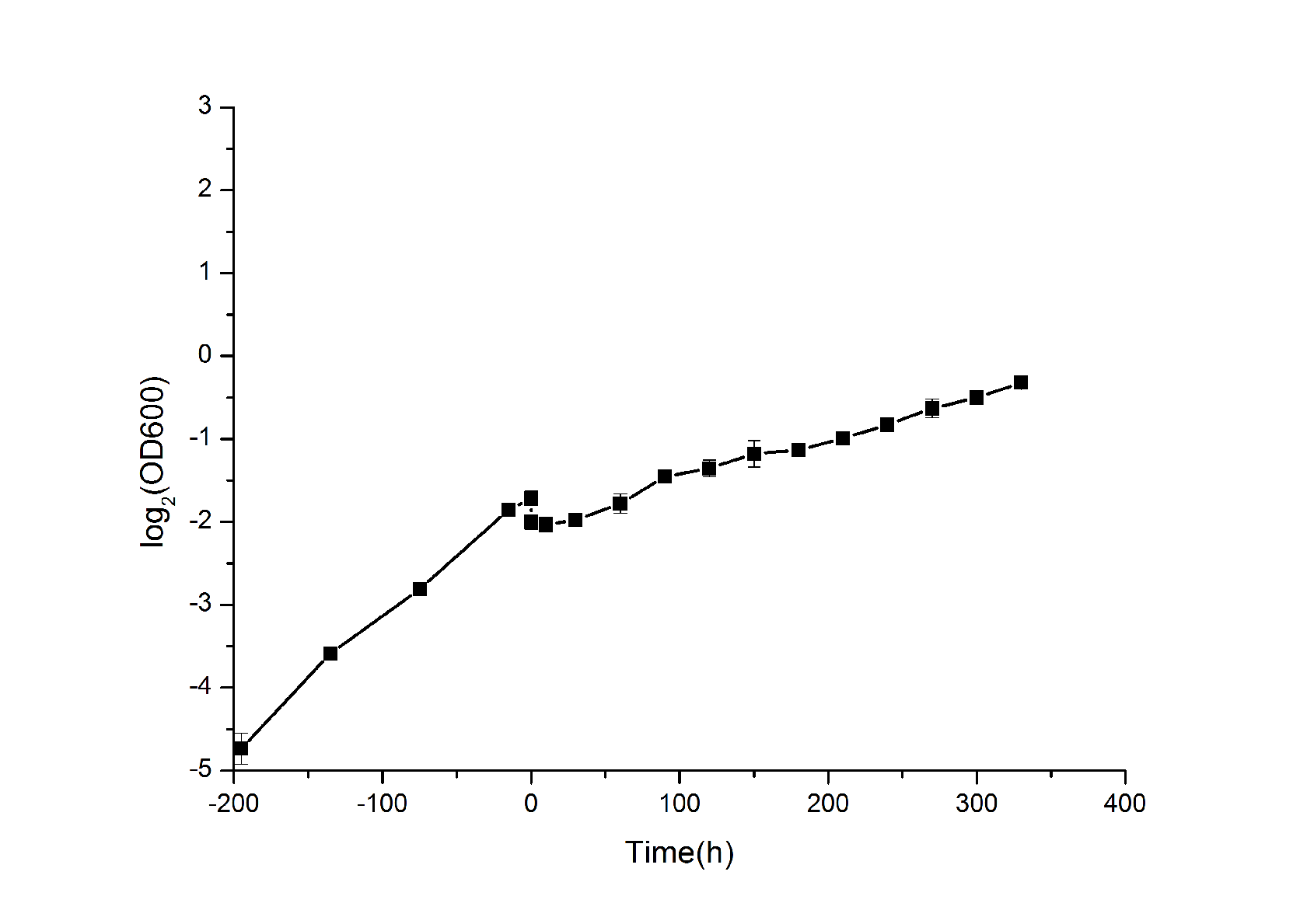

Supplement: S1 Fig — Bacteria were grown in Horikoshi-II medium with 0% NaCl until OD600 reached 0.3. Then, 32% (w/v) NaCl stock solution in Horikoshi-II medium was added up to the final concentration of 8% (w/v) NaCl, and samples were collected at 10, 30, 60, and 120 min after salt addition; the sample collected before salt shock (0 min) was used as a control. (TIF) (TIF) [file pone.0128649.s004.tif]

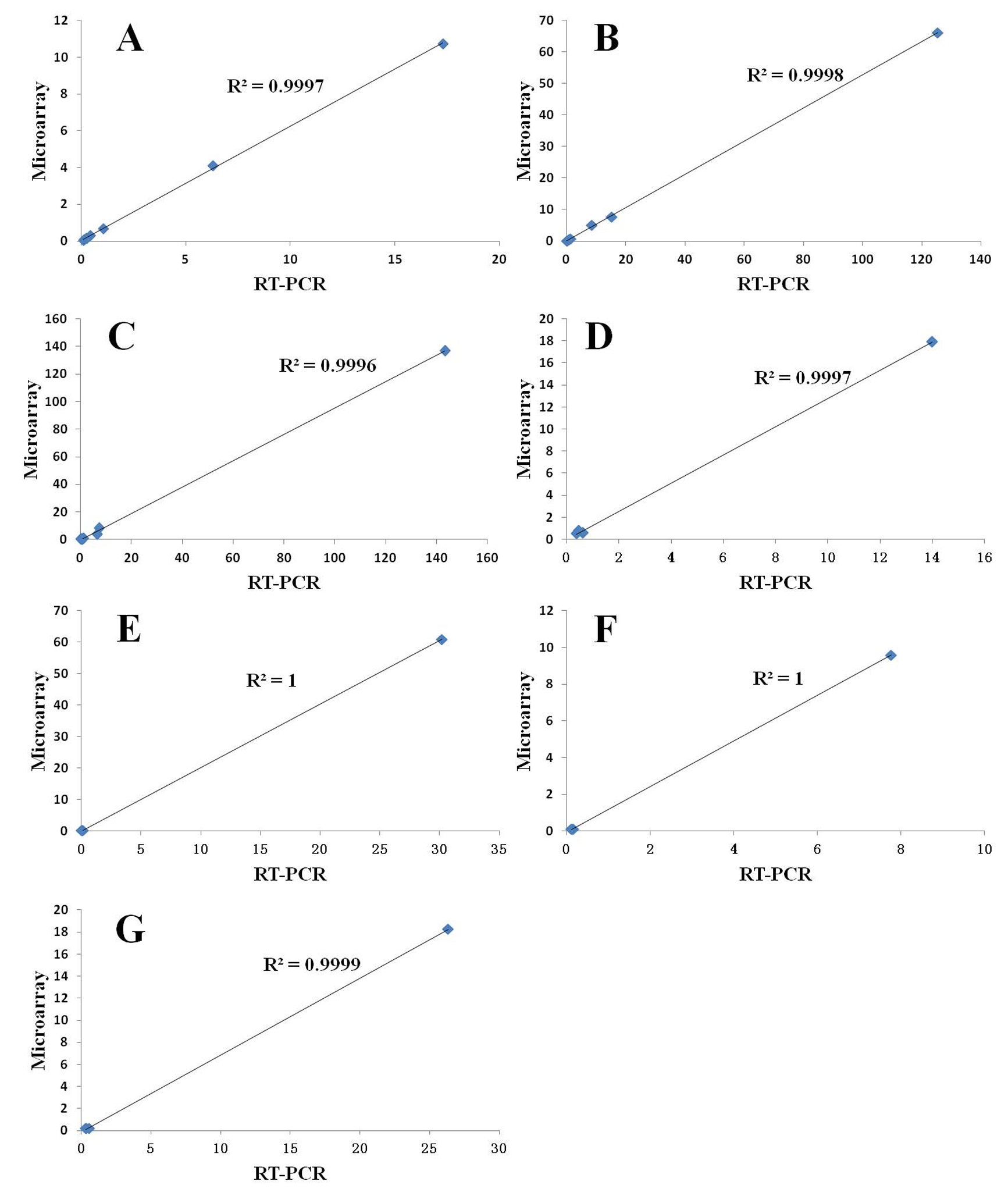

Supplement: S2 Fig — Eight genes were selected at random from differentially expressed genes and their expression levels were assessed by RT-PCR. (A) 2% vs 0%. (B) 8% vs 0%. (C) 15% vs 0%. (D) 10 min vs 0 min.(E) 30 min vs 0 min.(F) 60 min vs 0 min.(G) 120 min vs 0 min. (TIF) (TIF) [file pone.0128649.s005.tif]
