# Supplementary material for: Global Microarray Analysis of Alkaliphilic Halotolerant Bacterium Bacillus sp. N16-5 Salt Stress Adaptation
Source: PLoS One. 2015 Jun 1;10(6):e0128649. doi: 10.1371/journal.pone.0128649 (PMC4452262; doi:10.1371/journal.pone.0128649)
Supplement: S1 Table — (DOCX) (DOCX) [file pone.0128649.s006.docx]

**S1 Table The primers used for Quantitative RT-PCR amplifications.**

| **ProbeName** | **Primers** |
| --- | --- |
| 1 16S rDNA | 5' CCGTGGAGGGTCATTGGA 3' |
|  | 5' CGCCTCAGCGTCAGTTGT 3' |
| 2 orf2106 | 5' TGGTCATTGCGAAAGATAAAGTG 3' |
|  | 5' AGAATGGCGTGTAAGTCCCTG 3' |
| 3 orf3035 | 5' TAAAGAACTACGCCCACCAGA 3' |
|  | 5' TTATTGACCTCTTCTCCCATCG 3' |
| 4 orf2016 | 5' TTTATTGCTCGCATCTCACG 3' |
|  | 5' ACTTCATCCATTACAAGGGTCC 3' |
| 5 orf2667 | 5' TGCGTTAGCCACTGGTCTTAGC 3' |
|  | 5' GACAATCACGGTTACCACTTCTG 3' |
| 6 orf0321 | 5' GAAGGGTGTAGCAACCAAATG 3' |
|  | 5' TATCTAAGACTTTCAACCCAACCA 3' |
| 7 orf1862 | 5' GTAGGCCATACTGTGGAAGACC 3' |
|  | 5' TCCCTCATAGGACCTCGTTGC 3' |
| 8 orf1863 | 5’ AAGGCCAAAATAATTCAGCG 3' |
|  | 5' TCGTTTGAAGCATCACAGAGG 3' |
| 9 orf3034 | 5' TGAAGCAGAACTACGAGCAGC 3' |
|  | 5' TCCCGAACTCTATGGGCTAAG 3' |
