# Supplementary material for: Global Microarray Analysis of Alkaliphilic Halotolerant Bacterium Bacillus sp. N16-5 Salt Stress Adaptation
Source: PLoS One. 2015 Jun 1;10(6):e0128649. doi: 10.1371/journal.pone.0128649 (PMC4452262; doi:10.1371/journal.pone.0128649)
Supplement: S2 Table — (DOCX) (DOCX) [file pone.0128649.s007.docx]

**S2** **Table The primers used for construction of Δ*fur* strain**

| **ProbeName** | **Primers** |
| --- | --- |
| P1 | 5' ATTGGGATCCACCTCACCAGTATCCGACAGCAG 3' |
| P2 | 5' ATGACAAACTATTAAGTGGTGATCTGCGTACAGTAGCTTCTCGTTGA 3' |
| P3 | 5' TCAACGAGAAGCTACTGTACGCAGATCACCACTTAATAGTTTGTCAT 3' |
| P4 | 5' ATTGGTCGACAACCGAGGGCTGTATGCG 3' |
| P5 | 5' TCTTAATGAAAAACAACTAGAGA 3' |
| P6 | 5' CTTCTAGCATTGCTTTATTTCTCA 3' |
